# Supplementary material for: Causal Relationship between Aging and Anorexia Nervosa: A White-Matter-Microstructure-Mediated Mendelian Randomization Analysis
Source: Biomedicines. 2024 Aug 16;12(8):1874. doi: 10.3390/biomedicines12081874 (PMC11351342; doi:10.3390/biomedicines12081874)
Supplement: Supplementary file 1 [file biomedicines-12-01874-s001.zip › Supplementary Figure/Figure S9/Leaveoneout plot of MR analysis for ubm-b-1738 to AN.pdf]

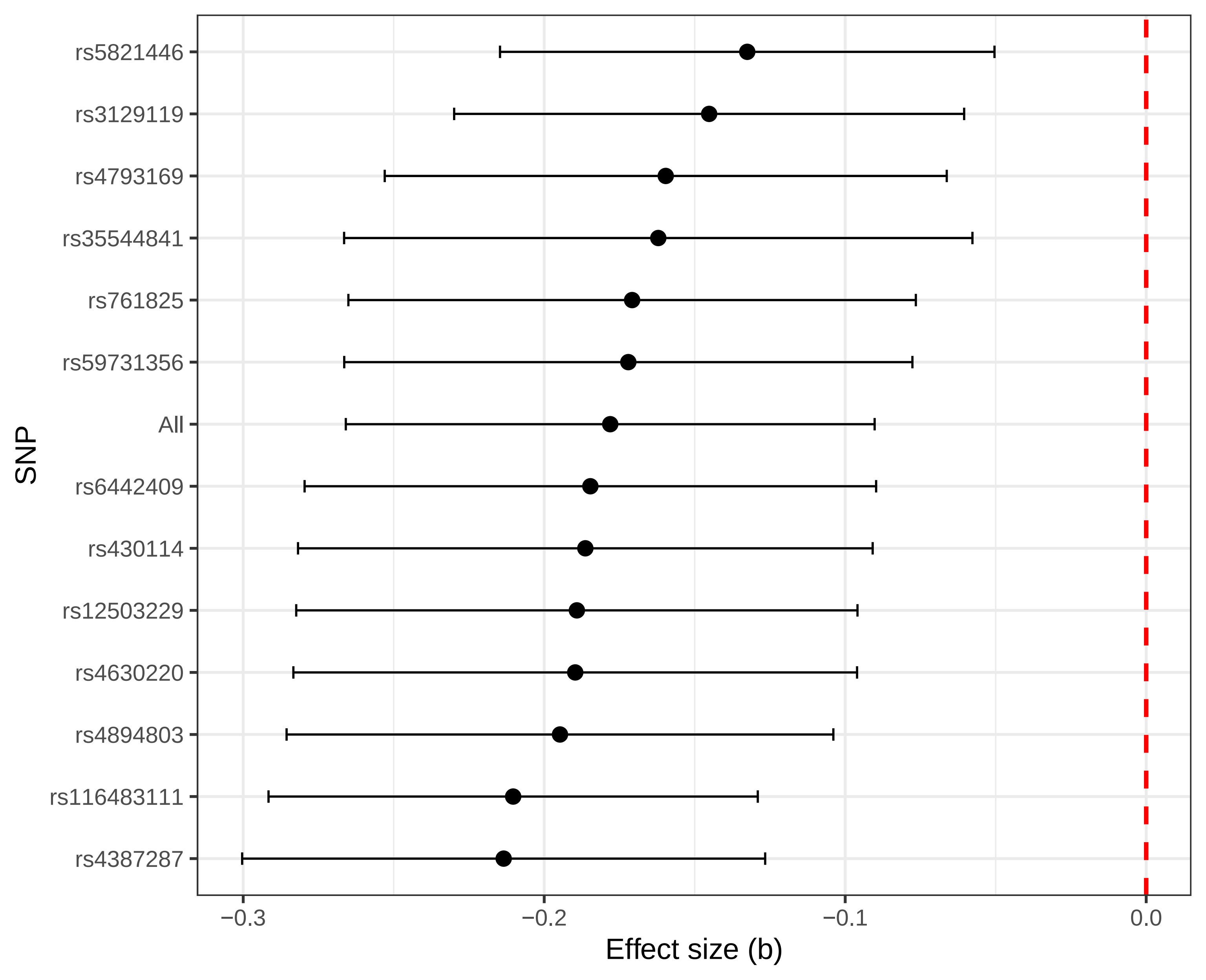

rs5821446
rs3129119
rs4793169
rs35544841
rs761825
rs59731356
All
rs6442409
rs430114
rs12503229
rs4630220
rs4894803
rs116483111
rs4387287
SNP
−0.3
−0.2
−0.1
0.0
Effect size (b)
